# Application of fourier transform infrared photoacoustic spectroscopy for quantification of nutrient contents and their plant availability in manure and digestate

**DOI:** 10.1016/j.heliyon.2024.e28487

**Published:** 2024-03-31

**Authors:** Khan Wali, Haris Ahmad Khan, Pietro Sica, Eldert J. Van Henten, Erik Meers, Sander Brunn

**Affiliations:** a Agricultural Biosystems Engineering Group, Wageningen University & Research, Wageningen, 6708 PB, Netherlands; b Data Science, Crop Protection Development, Syngenta, Basel, Switzerland; c Department of Plant and Environmental Sciences, Plant and Soil Science Section, University of Copenhagen, Copenhagen, Frederiksberg C 1871, Denmark; d Department of Green Chemistry and Technology, University of Gent, Gent, 9820, belgium

**Keywords:** Fourier transform mid-infrared photoacoustic spectroscopy (FTIR-PAS), Manure as a bio-based fertilizer, Assessment criteria, Partial least squares regression (PLSR, )

## Abstract

In this study, we assess the feasibility of using Fourier Transform Infrared Photoacoustic Spectroscopy (FTIR-PAS) to predict macro- and micro-nutrients in a diverse set of manures and digestates. Furthermore, the prediction capabilities of FTIR-PAS were assessed using a novel error tolerance-based interval method in view of the accuracy required for application in agricultural practices. Partial Least-Squares Regression (PLSR) was used to correlate the FTIR-PAS spectra with nutrient contents. The prediction results were then assessed with conventional assessment methods (root mean square error (RMSE), coefficient of determination R^2^, and the ratio of prediction to deviation (RPD)). The results show the potential of FTIR-PAS to be used as a rapid analysis technique, with promising prediction results (R^2^ > 0.91 and RPD >2.5) for all elements except for bicarbonate-extractable P, K, and NH_4_^+^-N (0.8 < R^2^ < 0.9 and 2 < RPD <2.5). The results for nitrogen and phosphorus were further evaluated using the proposed error tolerance-based interval method. The probability of prediction for nitrogen within the allowed limit is calculated to be 94.6 % and for phosphorus 83.8 %. The proposed error tolerance-based interval method provides a better measure to decide if the FTIR-PAS in its current state could be used to meet the required accuracy in agriculture for the quantification of nutrient content in manure and digestate.

## Highlights

• Utilizing FTIR-PAS for the prediction of macro- and micro-nutrients in a diverse range of manures and digestates.
• The prediction for total N was better with probability within the al-lowed limit (94.6%).
• The prediction for total P within the allowed limit was lower (83.8%).
• FTIR-PAS did not succeed in predicting bicarbonate-extractable P and NH_4_^+^−N.

## Graphical abstract

**Figure:**
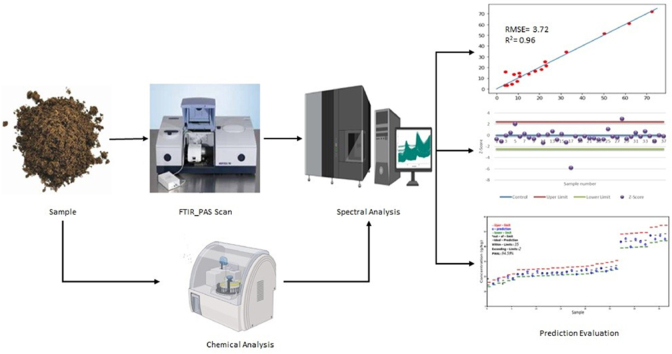

## 1 Introduction

The world's population continues to grow, resulting in increasing demand for food [1].To meet this demand, crop yields must be improved. One way to achieve this is by using more mineral fertilizers. However, as a result of this increased use of mineral fertilizers, soils have become more depleted of organic matter and other essential nutrients [2]. Moreover, the production of mineral fertilizers relies on the mining and processing of non-renewable resources, such as phosphate rock and potash. These resources are finite, and their extraction can be environmentally destructive, contributing to habitat destruction, soil erosion, and water pollution [3] [4,5],.

To counteract the negative effects of mineral fertilizers, modern agriculture is exploring the reuse of bio-based materials including livestock manure, composts, and digestate. These biowastes are alternatives that can be used to replace mineral fertilizers in agriculture. Bio-based materials contain essential macronutrients (N, P, K, S, Ca, and Mg), plant-available forms of nitrogen and phosphorus (Nitrate (NO_3_^−^-N) and NH_4_^+^-N, bicarbonate-extractable P) micronutrients (Na, Fe, and Zn) [[6], [7], [8]].

Although it is recognized that manure and digestate can increase crop yields, farmers are hesitant to rely on them due to concerns about the unknown nutrient composition [9]. Furthermore, the labile nature of nutrient content in bio-based materials poses a risk of over-application and environmental challenges [10]. Bio-based fertilizers also have variable concentrations of essential nutrients, depending on the animal, production system, and place where it was generated [11]. This is beneficial as different biowastes can be combined to meet a particular crop demand [11] Therefore, to avoid the risk of over- or under-application of one or more key elements, information of the composition of biobased fertilizers (concentration of nutrients and their plant-available forms) is needed.

For that purpose, traditional laboratory methods (wet chemical analyses) for nutrient quantification are commonly used to quantify nutrient contents. However, these methods are slow and expensive and are, therefore, inadequate for this purpose [12,13] An alternative method for the rapid quantification of nutrient content in bio-based materials (manure and digestate) is spectroscopy in the near-infrared (NIR) wavelength range (780–2500 nm) and mid-infrared (MIR) range (2500–4000 nm) [[14], [15], [16], [17], [18], [19]]. NIR spectroscopy is known for its weak absorption of overtone and combination bands, broadbands, and lack of distinct characteristics [20,21]. On the other hand, mid-infrared spectroscopy (MIR) has a greater potential for characterizing a wide range of samples due to its stronger absorption at fundamental frequencies and well-resolved spectral features that are linked to the components of the sample. However, as reported by Stumpe et al. [22], MIR is highly dependent on the particle size of the material. Additionally, MIRS typically has low resolution with very dark and opaque samples. To overcome this, a common solution is to dilute the sample with potassium bromide (KBr) powder to increase reflectance and prevent spectral distortion and non-linearities [23,24]. However, the use of KBr dilution in manure samples can be challenging due to the high organic content and potential variability in nutrient content. The high organic content of manure can interfere with the KBr absorption bands in the MIR spectrum, which can make it more difficult to accurately quantify specific nutrients.

Fourier transform infrared photoacoustic spectroscopy (FTIR-PAS), a combination of Fourier transform-infrared (FT-IR) and a photoacoustic detector (PA) is another alternative and can be used for the characterization of biowastes. FTIR spectroscopy involves the measurement of the infrared absorption spectrum by a sample, while PA spectroscopy involves the measurement of the acoustic pressure generated by the absorption of light by a sample. In recent years, the development of highly sensitive microphones has significantly increased the sensitivity of PA detectors and provided a way to overcome the limitations of traditional MIR. By combining FTIR and PA spectroscopy with appropriate statistical models, it is possible to achieve high levels of accuracy and precision in nutrient quantification in manure [25,26]. Additionally, the advantage of FTIR-PAS is that the shape of the photoacoustic spectrum is independent of the sample's morphology [27].

Bekiaris et al. [28]utilized FTIR-PAS for phosphorus speciation in digestates, while Huang et al. [29]applied it to determine the plant availability of phosphorus in digestate and digestate amended soil. However, to the best of our knowledge, for macronutrients and micronutrients in manure and digestates, the FTIR-PAS prediction capability is not investigated in the literature. Therefore, one objective of this research is to investigate the prediction capability of FTIR-PAS for essential macronutrients (N, P, K, S, Ca, and Mg), plant-available forms of nitrogen and phosphorus (NO_3_^−^-N, NH_4_^+^-N, and bicarbonate-extractable P) and micronutrients (Na, Fe, and Zn). Furthermore, while previous research has explored the use of spectroscopy sensors (Vis-NIR, MIR) for analyzing biowaste, our study, to the best of our knowledge, uniquely combines resources, encompassing manure from various animal sources (e.g., cow, pig, chicken) and digestates. This diversity of samples is not only from different animals but also from different sites.

A second objective of this study is to assess the prediction capabilities in view of the accuracy required for application in agricultural practice. In literature, a number of guidelines were proposed for the assessment of the prediction performance of spectroscopic sensing technologies, such as in Saeys et al. [30]and Zornoza et al. [31]. Commonly used indicators are, the coefficient of determination (R^2^) the root mean square error (RMSE), the ratio of performance deviation (RPD), the standard error of calibration (SEC), standard deviation (SD) [30,31]. As proposed by Saeys et al. [30], a value for R^2^ (0.66–0.80) indicates approximate quantitative predictions, whereas a value for R^2^ (0.81–0.90) means good prediction. Calibration models having R^2^ > 0.90 are considered to be excellent. According to Saeys et al. [30] and Zornoza et al. [31] RPD <2 is considered insufficient for applications, whereas a value for RPD between 2 and 2.5 makes approximate quantitative predictions possible. For values between 2.5 and 3 predictions can be classified as good, and an RPD >3 indicates an excellent prediction.

In view of the practical application of spectroscopic sensor technologies, two reflections are of interest. First of all, though R^2^, RMSE, and RPD are useful for model comparison and the selection and pre-treatment of spectral information, they do not provide information about the absolute goodness of fit or the predictive accuracy of the model. Moreover, R^2^, RMSE, and RPD do not provide information about the direction of error or error understanding in terms of easily interpretable units [[31], [32], [33], [34], [35], [36]]. Secondly, the limits for R^2^ > 0.9 and RPD >3, set by Saeys et al. [30]and Zornoza et al. [31] might not encourage the use of spectroscopic methods to replace wet chemical analysis methods in agricultural practices.

In this research, we will confront FTIR-PAS predictions with more practical accuracy standards. Oenema et al. , proposed the performance characteristics of NIR spectroscopy analysis results in order to maintain or improve compliance with and enforcement of the manure and ammonia policies at the current level. The report [37], clearly highlights the systematic and random error limits for nitrogen and phosphorus in accordance with prevailing ammonia policies. The procedure based on [37] was used by Derikx et al. [38] for the evaluation of NIR prediction for the measurement of total nitrogen (TN) and total phosphorus (TP).

The use of systematic and random error limits indicated in that report allows for the definition of error tolerance intervals to evaluate the predictive performance of FTIR-PAS for TN and TP in both manure and digestate. The error tolerance intervals could then be used to classify the prediction results in two classes i.e., acceptable and unacceptable, enabling us to quantify results in terms of percentage accuracy in view of application in agricultural practice.

The purpose of this study is to assess the prediction performance of Fourier transform infrared-photoacoustic spectroscopy (FTIR-PAS) for the determination of nutrient content and their plant availability in manure and digestate. Additionally, this study introduces a new tolerance-based error interval method to visualize the accuracy of prediction for TN and TP. To the best of our knowledge, this is the first time that the FTIR-PAS technique has been developed to quantify macro- and micro-nutrient content in manure and digestate and that errors are assessed in a more practically relevant way.

## 2 Materials and methods

### 2.1 Sample collection and chemical analysis

We collected 122 samples from different farms in the Netherlands and Belgium, consisting of cow manure, chicken manure, pig manure, and digestate. To minimize the available nitrogen ((NH_4_^+^-N) loss, zeolite (10 % of the available dry matter) was applied [39]. All the samples were then air dried at a temperature of 50^0^ for one week and then finely milled by a ball mill (Retsch PM 100) in order to make them powder. Afterward, the PrimacsSNC-100 analyzer was used for the determination of total nitrogen (TN) concentration in the samples. The concentration of P, K, Ca, Na, Fe, Mg, Zn, and S was determined by first digesting 300 mg of the sample with 5 % HNO_3_ in a closed microwave (CEM MARS 5, Belgium) followed by analysis using inductively coupled plasma optical emission spectrometry (ICP-OES) (Varian Vista MPX, United States) [40].

To determine the plant available forms of nitrogen (NH_4_^+^-N and NO_3_^−^-N), all samples were extracted with 1 M KCl (1:60 wv^−1^). After shaking for 60 min in the in-house made end-over-end shaker, samples were centrifuged using (Kubota model 5500) at 7000 rpm (2719 g) for 10 min and filtered through Whatman (number 5) filter papers [41]. The contents of ammonium (NH_4_^+^-N) and nitrate (NO_3_^−^-N) in the extract were measured by flow injection analysis (FIAstar 5000, Foss, Sweden). The NaHCO_3_ extractable P of biomaterials such as manure and digestate is highly correlated with plant availability [42]. A 0.5 M NaHCO$_3$ solution was prepared and the pH was adjusted to 8.5. All the samples were extracted by shaking for 30 min in the end-over-end shaker. After that, samples were centrifuged and passed through Whatman (number 5) filter papers. The orthophosphate content in the filtered extract was measured by flow injection analysis (FIAstar 5000, Foss, Sweden), using ammonium molybdate [43].

### 2.2 Fourier transform infrared photoacoustic spectroscopy (FTIR–PAS)

**Fig. 1:**
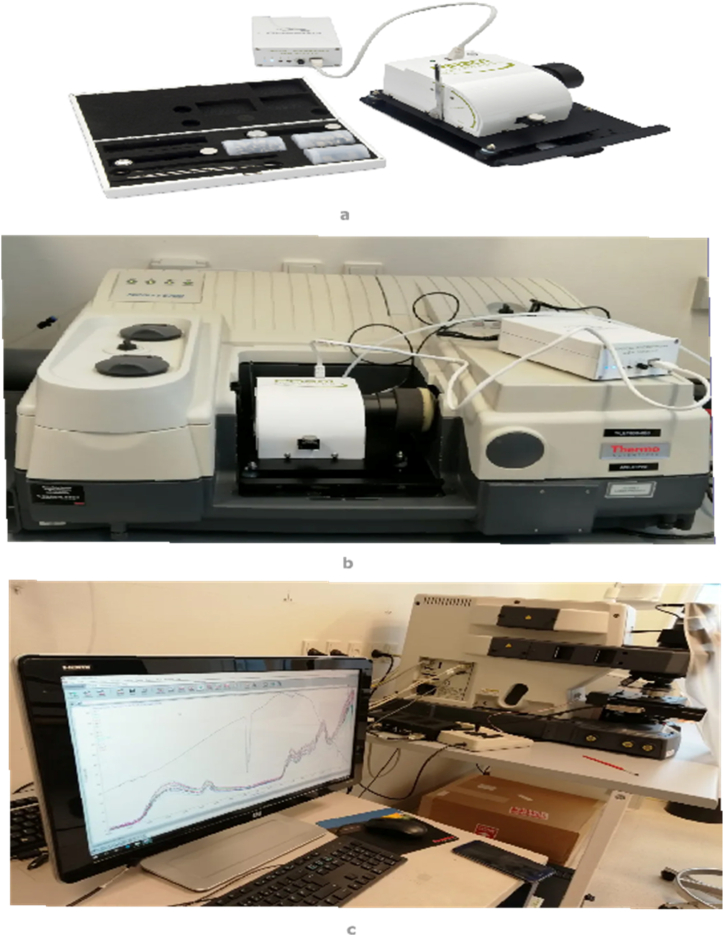
The experimental setup of FTIR-PAS scanning a) PAS detector b) FTIR mounted with PAS detector c) A computer monitor displaying the recoding of spectra of different samples.

The FTIR-PAS spectra were recorded using a Nicolet 6700 (ThermoScientific, USA) spectrometer equipped with a PA-301 photoacoustic detector (Gasera Ltd., Finland). The experimental configuration, including both the scanning procedure and the overall experimental setup, is depicted in Fig. 1a, b and 1c. A helium gas purging flow was used to reduce the noise produced by the evaporation of moisture from the samples. The samples were packed in small ring cups 10 mm in diameter. For each sample, 64 scans in the mid-infrared region between 4000 and 400 cm^−1^ at a resolution of 4 cm^−1^ were recorded and averaged. A spectrum obtained from a background sample was subtracted from the spectrum of each sample to eliminate the effect of ambient moisture and CO_2_. The standard carbon black background sample provided by Gasera Ltd. Was very porous and thin. During the spectral recording of certain samples, such as digestate, which had a dark appearance, there was a significant concern about the potential loss of a substantial amount of information, particularly in the region below 1000 cm^−1^ [28]. This might have happened most probably because the samples were not arranged as thin layers as in the reference. In order to eliminate this effect, a thicker layer of activated charcoal (Sigma-Aldrich Denmark ApS, CAS number: 7440-44-0) was used to record the background spectrum. The background was recorded after every 10th sample and subtracted from each sample spectra.

### 2.3 Sample characterization and statistical analysis

**Fig. 2:**
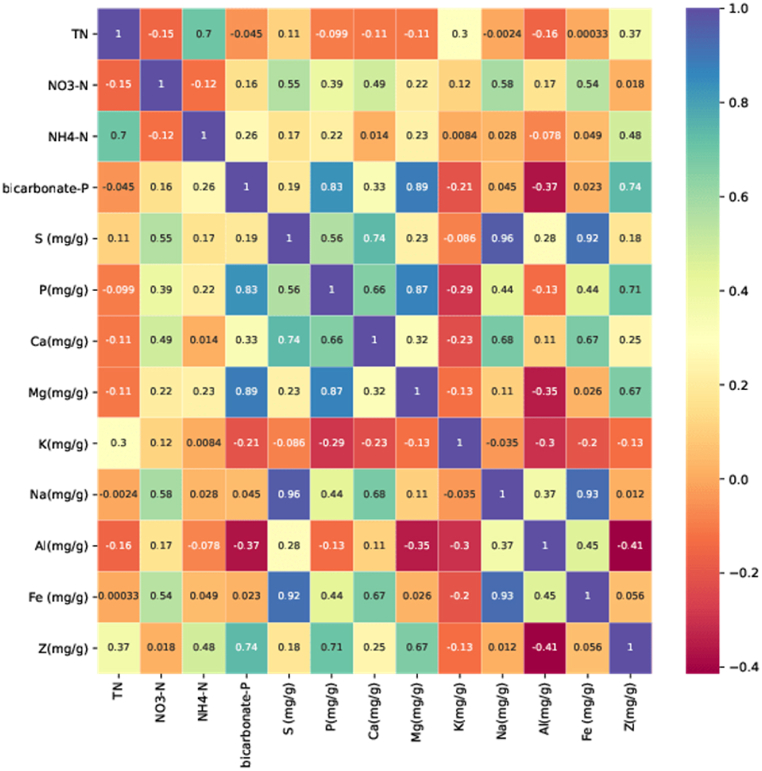
Correlation (r) matrix among the constituents as determined by wet chemical analysis.

The 12 chemical properties of interest presented in this paper belong to three broad categories. (1) Essential macronutrients (N, P, K, S, Ca, and Mg) (2) plant-available forms of nitrogen and phosphorus (NH_4_^+^-N and NO_3_^−^-N), bicarbonate-extractable P) (3) micronutrients (Na, Fe, and Zn). Most of the nutrients were highly correlated with each other as shown in Fig. 2. As expected NH_4_^+^-N and TN were highly correlated, while there was a weak correlation between TN and NO_3_^−^-N. This weak correlation might be due to the fact that the concentration of NO_3_^−^-N in the current samples was negligible. A strong correlation between TP and bicarbonate-extractable P, Mg, and Ca is also evident. The correlation between S, Ca, Na, Fe, Zn, and Fe was also high. This high correlation between different nutrients becomes very important for quantification using spectroscopy. The high correlation reduces the number of variables needed to be considered and increases the accuracy of the prediction. When multiple elements have a high correlation, they are influenced by the same underlying factors, and so a prediction for one element can provide information about the other elements as well. As a result, this leads to better prediction models. A high correlation between elements in a chemical analysis can also be used to validate spectral models. If the predictions made by the spectral model are consistent with the correlation observed between the elements, it provides additional evidence that the model is accurate and reliable [44]. Another advantage of strong correlations between the constituent variables makes it interesting to model the relationship between the variables of one group and the spectra at once using a partial least-squares regression for multiple variables [30]. This method reduces the noise sensitivity of the individual component calibrations compared to performing an independent PLS calibration for each component [45]. Though the high correlation between elements is helpful, it, however, presents the challenge of disentangling whether a correlation between independent variables and dependent variables is due to chance or an actual causal relationship (Cage of covariance) [46,47].

### 2.4 Data prepossessing

**Fig. 3:**
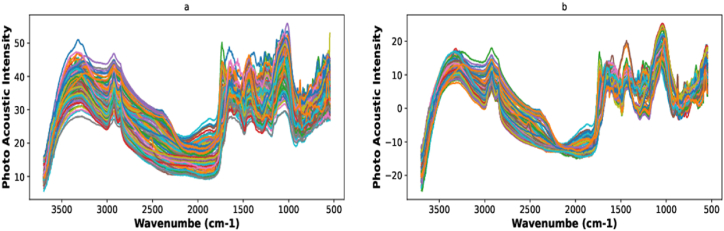
FTIR-PAS spectra a) Raw spectra b) after MS correction.

Pre-processing of FTIR-PAS spectra is considered an important part of any quantitative or qualitative analysis [48,49].Performing FTIR-IR spectroscopy is highly sensitive to background noise in the laboratory. This noise can reduce the signal-to-noise ratio (SNR) of the spectral information and, therefore, negatively affect a calibration model's accuracy. Other challenges associated with FTIR-PAS spectra include complex backgrounds and baselines, which introduce unwanted variations in the spectra and make calibration of the model complicated [50]. To deal with these problems the spectra are often pre-processed before performing any analysis. In the present study, FTIR-PAS spectral data were mean-centered followed by multiplicative scatter correction (MSC) as shown in Fig. 3a and b.

The scatter correction can help to remove any variations in the background signal that may interfere with the measurement of the analyte of interest [51]. This is particularly important in FTIR-PAS spectroscopy because the photoacoustic effect can cause additional noise and variations in the background signal, which can make it more difficult to accurately measure the sample [52]. No further pre-processing was performed as this might have a negative impact on the prediction performance [53,54].

### 2.5 Model development and optimization of PLS factor’

Partial least squares regression (PLS) is one of the most widely used multivariate prediction methods in chemometric analysis [55,56]. Partial Least Squares (PLS) regression is a suitable method for predicting the chemical composition of manure using spectral data because it can handle a large number of variables in the data and can identify the most important variables related to the response variable [19]. PLS is also less sensitive to collinearity and can be used for limited spectral data. PLS projects spectral data into latent variables that explain the variances within the spectral data. Given a spectral matrix X and the corresponding response variable Y, PLS is used to find the scores (T and U) with loading (P and Q) and error matrix (F) from the decomposition of X and Y as given in Equations (1), (2), (3)):

(1) $$X=TP^{\prime }+Fx$$

(2) $$Y=UQ^{\prime }+Fy$$

While the original space relation is:

(3) Y=XB+E

where matrix B is the regression coefficient and E is the residuals matrix. The goal of PLS is to build a regression model that can effectively predict the response variable based on a set of spectral data. For this purpose, the total data set (n = 122) was divided into a training set (70% i.e, n = 85) and a test set (30% i.e., n = 37) using train test split function from the scikit-learn library with random state = 1 [54]. The function randomly shuffles and partitions the dataset into two disjoint sets from each source of samples as shown in Table 1.

**Table 1:** Train test split from each source to ensure 70-30 % split.

Type	Total	Train	Test
Cow Manure	57	40	17
Digestate	28	19	9
Chicken manure	16	11	5
Pig manure	21	15	6

The number of PLS components used in the model is a key parameter that needs to be selected appropriately [57]. Using more factors improves the calibration, but it leads to poor results on test data, which is a sign of over-fitting. To get the best results, the number of factors should be optimized to minimize calibration and validation errors. To achieve this optimization, a common approach is to employ a technique known as leave-one-out cross-validation (LOOCV) [58]. LOOCV is a rigorous validation method where the model is repeatedly trained on all data points except one and tested on the omitted data point. This process is iterated for each data point, resulting in multiple model evaluations. LOOCV allows us to assess how well the model generalizes to new data points and helps identify the optimal number of PLS components. Therefore, the PLS regression model is constructed with varying numbers of.

**Fig. 4:**
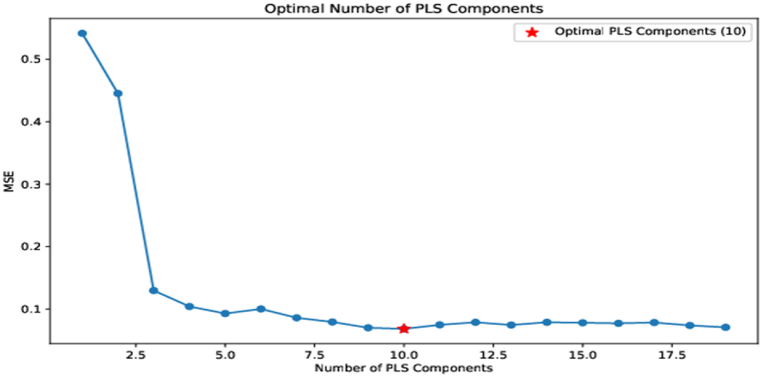
Optimization of PLS components using LOOCV.

Components. The model's performance is evaluated at each iteration of LOOCV, by calculating the mean square error (MSE) between the predicted and actual values. The optimized number of components is the one that has the lowest MSE during cross-validation as shown in Fig. 4. After the PLS optimization, the model was re-calibrated on the training set and then prediction was performed on the test data set.

### 2.6 Model assessment criteria

To evaluate the accuracy of predictions, three assessment criteria are compared.

#### 2.6.1 Traditional assessment criteria

The traditional performance parameters, root mean square error (RMSE), coefficient of determination R^2^, and the ratio of prediction to deviation (RPD) were used first to assess the results for each nutrient as in equations (4), (5), (6).

(4) $$RMSE=\sqrt{\frac{1}{n}\sum_{i=1}^{n}{\left(Y_{i}-{Y_{i}}^{\prime }\right)}^{2}}$$

(5) $$R^{2}=1-\frac{\sum_{i=1}^{n}{\left(Y_{i}-{Y_{i}}^{\prime }\right)}^{2}}{\sum_{i=1}^{n}{\left(Yi-\bar{Y}\right)}^{2}}$$

(6) $$RPD=\frac{STD\left(Y_{i}\right)}{RMSE}$$

Here ${Y_{i}}^{\prime }$ and $Y_{i}$ are the predicted and actual values of the response variables, $\bar{Y}$ is the mean value of the actual value of the response variable, and $STD\left(Y_{i}\right)$ is the standard deviation of the actual response variables. As stated in the introduction, these performance evaluations could be used to tune the prediction model and evaluate the prediction model performances against each other.

However, in order to make a decision if the results obtained from FTIR-PAS are suitable in view of the accuracy required for application in agricultural practices, it is not sufficient to rely solely on R^2^, RMSE, or RPD. To prove this hypothesis, additional evaluation methods are explored and explained in the next section.

#### 2.6.2 Evaluation of prediction using Z-score and confidence interval

Oenema et al. [37], proposed the performance characteristics of NIR spectroscopy analysis results in order to maintain or improve compliance with and enforcement of the manure and ammonia policy at the current level. The requirements for the accuracy of the determinations of nitrogen and phosphate via NIR spectroscopy for dried solid manure are set as given in Table 2.

**Table 2:** First phase requirements for the maximal systematic and random error for the determination of the N and P2O5 content of animal slurry by NIRS applied on manure tank (for 95% of the determinations) note: adopted from Ref. [34].

Element	Concentration (g kg^−1^)	Systematic error (g kg^−1^)	Random error (g kg^−1^)	σmax (g kg^−1^)
N	≤2.5 >2.5	<0.125 <5%	< ±0.5 < ±10%	0.25 0.1 YAct
P2O5	≤0.5 >0.5	<0.025 <5%	< ±0.15 < ±20%	0.075 0.15 YAct

The limits for random error and systematic error are adapted from CDM report Oenema et al. [37]. The CDM report used several aspects for the determination of the required accuracy; a)The possibilities for compliance and enforcement of the manure policy, b) The distinction between systematic errors and random errors, c) The distinction between solid manure and liquid manure, d) The reference method; flat rates or prescribed sampling and determination methods and, e) The technical-analytical possibilities and impossibilities. Therefore, the magnification coefficients for the maximum random error in the case of N and P_2_O_5_ are set at 0.1 and 0.15, respectively, to establish acceptable limits for these parameters.

The procedure based on [37]was used by Derikx et al. [38]for the evaluation of NIR prediction for the measurement of TN and TP. A Shewhart chart approach was used to monitor the test results. The chart displays the prediction results based on their Z-score, while the Z-score is calculated based on the following formulas (equation (7)):

(7) $$Z_{score}\left(i\right)=\frac{{Y_{i}}^{\prime }-{\;Y}_{i}}{\sigma_{\max}}$$

${Y_{i}}^{\prime }$ = NIR prediction result in g kg^−1^

$Y_{i}$ = Wet chemical analysis result in g kg^−1^

$\sigma_{\max}$ = Maximum allowed random standard deviation

$\sigma_{\max}$ was derived from the limits applicable to 95 % measurements. Initially, $\sigma_{\max}$, and then the 2 σ and 3 σ limits, were used based on the random error performance criteria set out in Table 2.

This method of evaluation of prediction results for TN and TP using FTIR-PAS was tested in the current study. All wet chemical analyses and the prediction results were transformed using the log_2_ transformation to obtain a normal distribution. Then the z-scores were calculated using equation (7). Evaluation using this method can provide a quick way to visually check the accuracy of the prediction. However, the method is based on calculating the Z-score, which requires the distribution of the data to be normal. Although we can use various normalization techniques to normalize the prediction results to a certain extent [59], it is not always possible to do so, especially if the samples come from different sources with varying levels of nutrient content, this can result in a non-normal distribution. In the present study, some of the elements could be transformed to a normal distribution by log_2_ transformation but other elements were always highly skewed. Therefore, an alternative method that is independent of the distribution type was required. One such method is to evaluate the prediction by defining the error tolerance-based intervals for each element of interest, as explained in the next section.

#### 2.6.3 Evaluation of prediction by error tolerance interval-based method

The proposed performance characteristics of NIR spectroscopy analysis results by Oenema et al. [37], and the requirements for the accuracy of determinations for nitrogen and phosphate for dried solid manure given in Table 2 were used to define intervals of acceptable error for TN and TP.

By comparing the prediction results to this range, we can determine how often the results fall within an acceptable limit. This method of visualization can provide an alternate way of interpreting the prediction results. Therefore, the acceptable limits are defined using the following equations (8), (9)):

(8) $$ALL\left(i\right)={\;Y}_{i}\;-\;\epsilon \left(i\right)$$

(9) $$AUL\left(i\right)={\;Y}_{i}+\epsilon \left(i\right)$$

ALL(i) = Acceptable Lower limit for ith prediction

AUL(i) = Acceptable Upper limit for ith prediction

ϵ(i) = The maximum random error allowed as per Table 2

The value of ϵ(i) according to Table 2 is constant when the concentration is less than 2.5 g kg^−1^ for nitrogen. When the concentration is greater than 2.5 g kg^−1^, it is chosen to be 0.1 times the actual values according to Table 2. The ϵ(i) value for the other elements can be found according to some standard as to how much tolerance is acceptable in the application. We can get a good indication of the accuracy of the predictions from the plots and the number of predictions within the allowed limits. The evaluation of prediction results using this method is independent of the type of distribution and does not suffer from outlier effects. The results can also be visually analyzed to find the direction of error and whether the model is over or under-predicting the response variable.

## 3 Results

### 3.1 FTIR-PAS spectra of bio-materials

The FTIR-PAS spectrum is shown in Fig. 3. Clear peaks at various regions are visible in several spectral regions, particularly around 2800–3500 cm^−1^, 2200–2600 cm^−1^, 1800–2100 cm^−1^, 1700–900 cm^−1^ and 900-500 cm^−1^. The peaks at different regions were compared with those in literature to extract useful information about the chemical composition of bio-materials (manure and digestate) [25,[60], [61], [62]]. There are more peaks in the region below 1300 cm^*−*1^ which is an indication of strong information about phosphorus species according to Bekiaris et al. [20]. According to Changwen et al. [63],calcium carbonate should show peaks in the regions of 3100–2900 cm^*−*1^, 2600–2300 cm^*−*1^, 1600–1000 cm^*−*1^, 1700–1600 cm^*−*1^ and 2200–2100 cm^*−*1^ in soil.

In the current study strong peaks were observed in the region between 3100 and 2900 cm−1. While other strong peaks were observed at 2000-1600 cm^*−*1^ and the absorption in the regions 1600–1000 cm^*−*1^ was very strong but was heavily interfered with by some other absorption. There are a number of peaks observed in the range 900-500 cm^*−*1^. The Broad region at 3400 cm^*−*1^ primarily corresponds to the stretching of O–H and N–H, 2850–2926 cm^*−*1^ for Aliphatic C–H stretching, 1700-1600 cm^*−*1^ Carbonyl (C=O), 1440 cm^*−*1^ Methylene (CH2), 1365 cm^*−*1^ Amide I (C=O stretching of amides), 1090 cm^*−*1^ Amide II (N–H bending of amides), 1050 cm^*−*1^ Amide III (C–N stretching of amides), 895-600 cm^*−*1^ Inorganic anions (Cl, SO4, CO3) [25,60,61,64].

### 3.2 Prediction results using R^2^, RMSE and RPD

The prediction results based on FTIR-PAS on the test data set are presented in Table 3. Table 3 shows the assessment criterion parameters R^2^, RMSE, and RPD values for each nutrient. It can be seen that the prediction results for TN, TP, Ca, S, Mg, Na, and Fe are superior to those of NH_4_^+^-N and Bicarbonate-P. The prediction of K was not promising using FTIR-PAS in the current study, this could be the result of the low sensitivity of K in photoacoustic absorption. Changwen et al. [63]also concluded that the poor prediction of K using FTIR-PAS is because of the less sensitive nature of K in FTIR-PAS absorption. However, in comparison with the literature the R^2^ = 0.84 and RPD = 2.8 would lie within the range of good prediction as suggested by Saeys et al. [18]. The prediction results for plant available N (NH_4_^+^-N) are also in the acceptable range according to Saeys et al. [30]having R^2^ = 0.85, RMSE = 0.98 and RPD = 2.7. The prediction of TP having R^2^ = 0.93, RMSE = 1.96 and RPD = 4.01 can also be classified as better performance according to Saeys et al. [30].

**Table 3:** Partial least-squares regression prediction results for all the nutrients on test data.

Element	Range	Mean R^2^ RMSE RPD			
	(mgg^*−*1^)	(mgg^*−*1^)		(mgg^*−*1^)	
Total N	13.2–44.12	25.16	0.97	1.74	5.46
NH_4_–N	0–18.63	2.57	0.84	0.99	2.54
Total P	0–30	10.67	0.93	1.96	4.01
Bicarbonate-P	1.09–15.62	4.8	0.80	1.59	2.34
K	0–60	18	0.83	4.7	2.24
Ca	4.4–90	19.4	0.96	3.4	4.68
Mg	2.3–28	7.7	0.92	1.22	4.61
Na	0.1–21	4.14	0.92	1.46	3.06
S	1.4–15	5.38	0.95	0.88	4.25
Zn	0.0–0.67	0.18	0.95	0.037	4.45
Fe	0.3–13.1	2.47	0.91	1.1	2.82

The prediction results for Ca, Mg, Fe, Zn, Al, and Na fall within the excellent range (with R^2^ > 0.9 and RPD >2.9) as suggested by Saeys et al. [30]. By evaluating the results using R^2^, RMSE, and RPD, the results for all elements in the current study appear to be in the acceptable to excellent range, and FTIR-PAS is proven to be an alternate method of nutrient quantification according to these assessment criteria. However, from a practical perspective, whether the FTIR-PAS spectroscopy prediction capabilities meet the accuracy required for its application in agricultural practice, these assessment criteria are not enough. A value of R^2^ > 0.9 and RPD >2.5, does not always guarantee that the sensor could be used as a replacement for wet chemical analysis.

### 3.3 Prediction results using Z-score

**Fig. 5:**
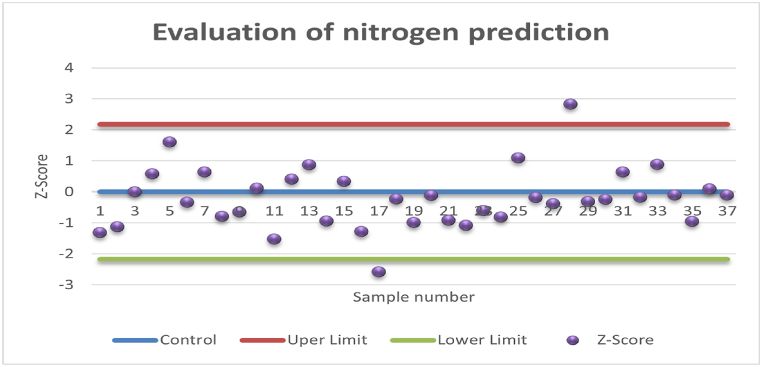
Evaluation of prediction results of nitrogen using Z-score.

**Fig. 6:**
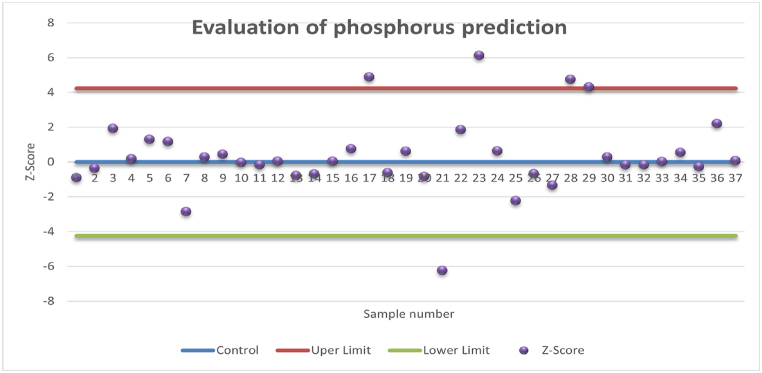
Evaluation of prediction results of phosphorus using Z-score.

The prediction results using the Z-score for nitrogen and phosphorus are shown in the form of the Shewart chart in Fig. 5, Fig. 6. As proposed by Derikx et al. [38], the Z-score and Shewart chart approach was used to visually inspect the result by defining a tolerance error interval. This method is beneficial and the results for total nitrogen and total phosphorus were evaluated using this method by using the error margins given in Table 2. The actual and predicted values were first transformed using log_2_ transform and then the Z-Scores were calculated using equation (7). Approximately 95 % of the time the prediction for nitrogen prediction lies within an acceptable range, while for phosphorus, 86 % of the prediction lies within the allowed limits.

### 3.4 Prediction results using the error tolerance-based interval method

**Fig. 7:**
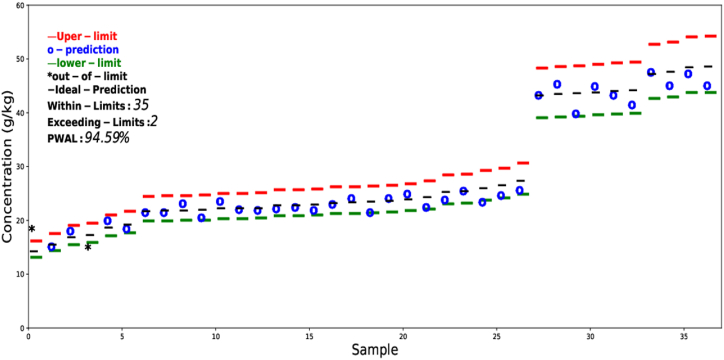
Prediction results based on the error tolerance-based interval method for total nitrogen arranged in ascending order.

**Fig. 8:**
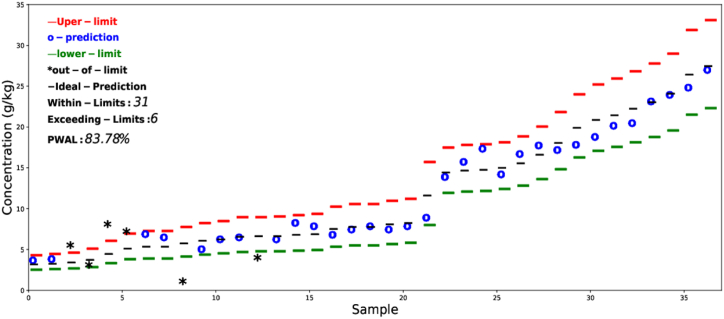
Prediction results based on the error tolerance-based interval method for total phosphorus arranged in ascending order.

The prediction results using the error tolerance-based interval method are presented in Fig. 7, Fig. 8. For better visualization, the results are arranged in ascending order. The current work only presents the prediction results for total nitrogen and phosphorus. The allowed limits were calculated based on Table 2. The results show that for nitrogen 35 out of 37 and for phosphorus 35 out of 37 prediction lies within the allowed limit. The probability that the prediction for nitrogen to fall within the allowed limit is 94.6 % while that of phosphorus is 83.8 %. The error tolerance-based interval method could be used for other nutrients and chemical properties as well but was excluded from the current study as there are no allowed limits for other elements reported in the literature. Further work is needed to define how much error could be tolerated for other chemical properties.

## 4 Discussion

The potential use of FTIR-PAS as an alternate method for the estimation of nutrient content in livestock manure and digestate was evaluated. FTIR-PAS was able to predict the total nitrogen with the highest (R^2^ = 0.97 and RPD = 5.46) compared to other elements in the current study. The FTIR-PAS prediction for all other nutrients (P, Ca, Mg, Zn, Na, Fe, and Al) was also better. The prediction results were lower for NH_4_^+^-N, bicarbonate-extractable P, and K. Huang et al. [29] also reported FTIR-PAS prediction with the same performance for plant available P in digestates. The lower prediction capability of FTIR-PAS for K could be the result of the low sensitivity of K in photoacoustic absorption [63]. Therefore, its prediction could be the result of its correlation with other spectral active chemical components such as nitrogen. The prediction results for NH_4_^+^-N were also poor despite the fact that it is highly correlated with total nitrogen. The drying process could be potentially one of the reasons for the poor NH_4_^+^-N prediction. This is because during the drying of the samples, most of the NH_4_^+^-N was lost and the concentration of NH_4_^+^-N was very small (mean value 2.4 g kg^−1^). It has been reported in our previous study [19]that NIR and MIR prediction of TN and NH_4_^+^-N at low concentrations were poorer compared to high concentrations. The same results were observed in the current study. Another reason for the low prediction of some elements might be due to the nature of the samples. As the samples were collected from different farms in different locations across the Netherlands and Belgium, as a result, there is a huge difference in the concentration of different elements. This might have made the calibration difficult and resulted in a poor prediction. The performance could be improved using careful selection of samples. The literature on application of spectroscopy techniques, showed that models calibrated at a particular site perform better on test set from the same site [65]. However prediction performance reduces if the test set is collected from a different site. Therefore, local calibration or site specific calibration is expected to perform better. Overall, the prediction results if evaluated according to traditional assessment criteria, the obtained results are promising ((with R^2^ > 0.9 and RPD >2.5)) [30]. However, when it comes to the application of spectroscopy and making a decision on whether the FTIR-PAS spectroscopy prediction capabilities meet the accuracy required for its application in agricultural practices, and can be used as an alternate method, R^2^ and RPD do not provide enough information [[34], [35], [36]].

To overcome the challenges associated with traditional assessment criteria, the prediction evaluation using the Z-score and the error tolerance-based interval method are introduced. Using Z-score, the prediction results can be easily visualized. The Z-score and tolerance intervals are calculated using equation (7) and Table 2. To account for 95 % data, the upper and lower limits are calculated as 1.96 times the standard deviation of the Z-score. The evaluation of the nitrogen prediction using Z-score as shown in Fig. 5, suggests that only two sample predictions are outside of allowed limits. For the case of phosphorus, the prediction results are poor, and five sample predictions are out of the allowed limit. Although FTIR-PAS has been reported in the literature for speciation of phosphorus [20,29], when it comes to its quantification, the results in the present study are not in the acceptable range.

These results suggest that further investigation is needed to explore other chemometric and pre-processing methods, which could potentially improve the prediction performances of FTIR-PAS. The evaluation of results using the Z-score provides useful information about the accuracy of prediction results, however, the data need to be carefully analyzed before using this method. As the Z-score method is only useful when the data is normally distributed, and transformation to the normal distribution is not always effective [66]. Therefore, to overcome these problems, an error tolerance-based interval method was introduced.

The error tolerance-based interval method is independent of the distribution type of the data and can provide both visual and quantitative information about the predicted results. The allowed tolerance error limits in the current study were defined in accordance with Table 2 using equations (8), (9)). The ϵ(i) value for nitrogen is always chosen 0.11 times the actual value to ensure ±10 % allowed error. It can be observed from Fig. 7, that at concentrations greater than 20 g kg^−1^, all the predictions lie within the allowed limit. The same conclusion can be derived for phosphorus, all the over-estimated values are at lower concentrations of phosphorus. As the concentration of phosphorus increases, the prediction results start to fall within the allowed limit. This method provides all the necessary information regarding the accuracy of prediction by dividing the results into two classes. For the case of nitrogen, overall good accuracy was observed as 94.6 % of the prediction lies within the allowed tolerance interval, while for phosphorus, 83.8 %.

In the current work, only nitrogen and phosphorus prediction results were analyzed using the tolerance-based error interval method as shown in Fig. 7, Fig. 8, as there was a clear indication of how much error can be tolerated for the application of manure as a fertilizer. Further work is needed for secondary nutrients (Ca, Mg, S, Zn, K) to find the tolerance intervals. The results based on both conventional and tolerance-based error intervals in the current suggest that FTIR-PAS have the potential to be used as a tool for rapid determination of nutrient content in bio-materials such as manure and digestate. Although the prediction results for nitrogen and phosphorus have high R^2^ and RPD values, all the results were not within an acceptable range. It should be noted that in the current study, the samples were collected from not only different sources but also from different locations. The prediction performance of FTIR-PAS could be improved if the calibration is done on one specific farm, thus could potentially replace the wet chemical analysis on-site.

## 5 Conclusions and recommendations for future research

The study demonstrated FTIR-PAS as a rapid analytical technique to quantify the nutrient content in a diverse set of bio-materials containing manure from different animals and digestate to be later used as fertilizers. Furthermore, the challenges associated with the conventional assessment guidelines for the prediction of the nutrient content are highlighted and a new error tolerance-based interval method is proposed. The proposed tolerance-based interval method helped in assessing the accuracy in a more practical way. The results for all elements have a high value of R^2^ > 0.9 and RPD >2.5. However, if evaluated in a practical scenario, the results were not within the allowed limits. The probability that the prediction falls within the acceptable range of accuracy for nitrogen was 94.6 % while for phosphorus 83.8 %. Due to the availability of tolerance level of error for the prediction of nitrogen and phosphorus, only these elements are investigated using the newly proposed method. Therefore, further work is needed to define the tolerance intervals for other nutrients and chemical properties. From the prediction results, it is evident that FTIR-PAS in combination with the PLS model is highly effective, particularly in predicting the concentration of nitrogen and phosphorus. However, more research is needed to improve the prediction performance of FTIR-PAS in order to meet the accuracy required for its application in agricultural practices. Therefore, in the future, we will investigate advanced machine learning techniques and explore more pre-treatment methods to achieve better accuracy of prediction. The prediction performance of FTIR-PAS could be further improved if the calibration is done on one specific farm, thus potentially meeting the required performance**.**

## Funding

This study has been developed within the frame of the European Union's 10.13039/501100007601Horizon 2020 research and innovation program under the Marie Sklodowska- Curie grant agreement No. 860127.

## Data availability

Data will be made available on request.

## CRediT authorship contribution statement

**Khan Wali:** Writing – review & editing, Writing – original draft, Visualization, Validation, Software, Methodology, Investigation, Formal analysis, Data curation, Conceptualization. **Haris Ahmad Khan:** Writing – review & editing, Supervision, Software, Formal analysis. **Pietro Sica:** Writing – review & editing, Formal analysis, Data curation. **Eldert J. Van Henten:** Writing – review & editing, Writing – original draft, Supervision, Project administration, Formal analysis. **Erik Meers:** Writing – review & editing, Supervision, Formal analysis. **Sander Brunn:** Writing – review & editing, Supervision, Formal analysis.

## Declaration of competing interest

The authors declare that they have no known competing financial interests or personal relationships that could have appeared to influence the work reported in this paper.
