# Supplementary material for: Application of fourier transform infrared photoacoustic spectroscopy for quantification of nutrient contents and their plant availability in manure and digestate
Source: Heliyon. 2024 Mar 31;10(7):e28487. doi: 10.1016/j.heliyon.2024.e28487 (PMC11002050; doi:10.1016/j.heliyon.2024.e28487)
Supplement: Multimedia component 1 [file mmc1.docx]

|  | Prediction Results over training and test data | | | | | | |
| --- | --- | --- | --- | --- | --- | --- | --- |
|  |  | Training set | | | Test set | | |
| Element | LVs | R^2^ | RMSE | RPD | R^2^ | RMSE | RPD |
| Total N | 10 | 0.98 | 1.52 | 6.12 | 0.97 | 1.74 | 5.46 |
| NH_4_^-^ -N | 13 | 0.88 | 0.93 | 2.98 | 0.84 | 0.99 | 2.54 |
| Total P | 10 | 0.96 | 1.77 | 4.34 | 0.93 | 1.96 | 4.01 |
| Bicarbonate-P | 12 | 0.87 | 1.42 | 2.78 | 0.80 | 1.59 | 2.34 |
| K | 11 | 0.90 | 3.2 | 2.49 | 0.83 | 4.7 | 2.24 |
| Ca | 13 | 0.97 | 2.9 | 4.96 | 0.96 | 3.4 | 4.68 |
| Mg | 10 | 0.94 | 1.07 | 5.13 | 0.92 | 1.22 | 4.61 |
| Na | 10 | 0.94 | 1.28 | 3.57 | 0.92 | 1.46 | 3.06 |
| S | 11 | 0.97 | 0.68 | 4.72 | 0.95 | 0.88 | 4.25 |
| Zn | 12 | 0.98 | 0.013 | 4.88 | 0.95 | 0.037 | 4.45 |
| Fe | 12 | 0.95 | 0.89 | 3.11 | 0.91 | 1.1 | 2.82 |
